# Risk factors for abnormal bone mineral density in Duchenne muscular dystrophy and the association of regular rehabilitation with bone health

**DOI:** 10.3389/fped.2026.1840046

**Published:** 2026-08-27

**Authors:** Yi Chen, Shaowei Dong, Meihuan Huang, Guixiang Liu, Turong Chen, Chunming Zhou, Mingsheng Zhang

**Affiliations:** 1 Department of Physical Medicine and Rehabilitation, Guangdong Geriatric Institute, Guangdong Provincial People’s Hospital (Guangdong Academy of Medical Sciences), Southern Medical University, Guangzhou, China; 2 Department of Research, Shenzhen Children's Hospital, Shenzhen, China; 3 Department of Rehabilitation Medicine, Shenzhen Children's Hospital, Shenzhen, China; 4 Department of Physical Medicine and Rehabilitation, Gaozhou People's Hospital, Maoming, China

**Keywords:** bone mineral density, children, Duchenne muscular dystrophy, mediation analysis, rehabilitation, risk factors

## Abstract

**Background:**

We investigated the prevalence and risk factors of abnormal bone mineral density (BMD) in Duchenne muscular dystrophy (DMD) and explored the potential association of regular rehabilitation with BMD.

**Methods:**

A cross-sectional study including 215 boys with DMD was conducted. Multivariate logistic regression was used to identify independent predictors, and mediation analysis was performed to explore the pathway linking rehabilitation and BMD.

**Results:**

Abnormal BMD was found in 62 patients (28.8%). Age was the strongest risk factor (adjusted OR = 1.0533 per month, 95%CI 1.0318–1.0752, *P* < 0.001), and regular rehabilitation was independently associated with a lower risk of abnormal BMD (adjusted OR = 0.2369, 95%CI 0.0935–0.6001, *P* = 0.002). The model showed good performance (Nagelkerke *R*^2^ = 0.591, AUC = 0.906). Muscle function mediated 15.7% of the association.

**Conclusion:**

Regular rehabilitation was associated with 76.3% lower risk of abnormal BMD in DMD patients, suggesting a potential beneficial association. Age remained the predominant non-modifiable risk factor. Better muscle function partially mediated the favorable association between rehabilitation and BMD. These findings support integrating regular rehabilitation into routine care for DMD.

## 1 Introduction

Duchenne muscular dystrophy (DMD) is an X-linked recessive neuromuscular disorder that affects approximately 1 in 3,500–6,000 live male births (1). The condition arises from pathogenic variants in the DMD gene, leading to progressive muscle degeneration with eventual loss of ambulation (2). Despite recent progress in targeted therapies, long-term glucocorticoid (GC) treatment remains the mainstay of management.

Over the past 50 years, multidisciplinary care including glucocorticoid therapy, non-invasive ventilation, cardiac care, and structured rehabilitation has improved median survival from 18.2 years among those born before 1970 to 24.0 years in contemporary cohorts (3, 4). As survival has improved, clinical attention has shifted to long-term comorbidities. Reduced bone mineral density (BMD) and associated fragility fractures represent a major clinical problem, affecting 21%–44% of adolescents and contributing to irreversible functional decline (5, 6).Notably, fat embolism syndrome complicates 1%–22% of these fracture cases (6). This severe complication further underscores the clinical urgency of standardized bone health management for paediatric DMD population. Multiple factors contribute to bone loss in this population, including glucocorticoid-induced suppression of osteoblast activity, reduced mechanical loading due to immobility, and prevalent nutritional deficiencies. However, the independent effects and interactions of these factors remain poorly defined (7–9).

Several important limitations exist in the current literature. Most studies are small, which limits investigation of interactions among variables, such as the combined effects of rehabilitation and ambulation on BMD, and reduces generalizability (10, 11). Although rehabilitation may help reduce bone loss, the optimal intensity and timing of such interventions remain uncertain (12, 13). The influence of genotype on skeletal health has also not been fully clarified, and few studies have directly examined the relationship between motor function measured by the Motor Function Measure (MFM) and bone health.

These gaps matter more than ever as patients with DMD increasingly survive into adolescence and adulthood. Skeletal complications have become a central focus in pediatric and transitional care (14), yet current clinical guidelines lack strong evidence-based recommendations for monitoring and managing BMD. What we need are larger studies with detailed phenotypic data to better identify at-risk patients and address modifiable risk factors.

The present study aims to fill these research gaps by investigating the prevalence and risk factors for abnormal BMD in a large cohort of boys with DMD, and by exploring the potential link between regular rehabilitation and bone health. Specifically, we aim to (1) quantify the prevalence of low BMD among Chinese boys with DMD; (2) identify independent risk factors for low BMD, with particular attention to modifiable factors such as rehabilitation participation; and (3) examine whether muscle function mediates the relationship between rehabilitation and BMD. Findings from this study are expected to inform targeted bone health strategies, including early prevention and personalized rehabilitation regimens, to improve long-term clinical outcomes for “patients with DMD.”

## 2 Methods

### 2.1 Study design and participants

This was a single-center, retrospective, cross-sectional study conducted at the Department of Rehabilitation Medicine, Shenzhen Children's Hospital. We enrolled boys with a confirmed diagnosis of Duchenne muscular dystrophy (DMD) who were treated at our institution between January 2020 and November 2025. For this analysis, we only used data from the initial assessment (first visit) to maintain a cross-sectional design.

The study was approved by the Ethics Committee of Shenzhen Children's Hospital [Registration No. SZEH-IRB(Research) 202303202], which waived the requirement for informed consent.

**Inclusion criteria:**

1. Male sex
2. Age 3–18 years at enrollment
3. Confirmed DMD diagnosis by genetic testing or muscle biopsy
4. History of glucocorticoid treatment

**Exclusion criteria:**

1. Other neuromuscular or neurodevelopmental disorders
2. Comorbidities that independently affect bone health (e.g., osteogenesis imperfecta, cerebral palsy, thyroid dysfunction)
3. Current use of bone-active medications (e.g., bisphosphonates)
4. Previous treatment with gene therapy

**Figure 1:**
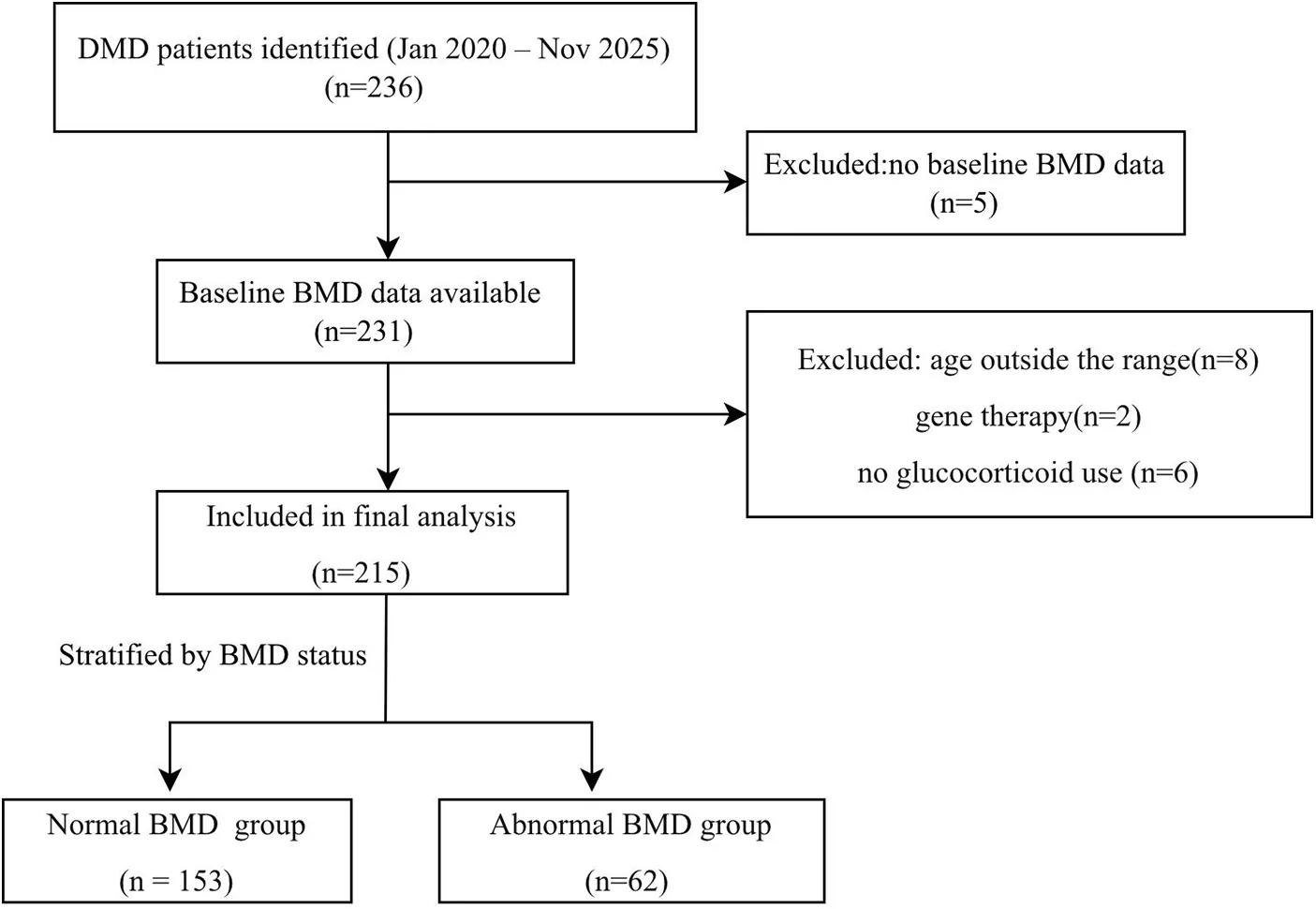
Flowchart of patient selection. None of the 215 assessed participants met the exclusion criterion for current use of bone-active medications (e.g., bisphosphonates).

A flowchart of patient selection is presented in Figure 1.

### 2.2 Data collection

#### 2.2.1 Standardized data extraction

Medical records were systematically reviewed to collect demographic, clinical, laboratory, imaging, anthropometric, and functional rehabilitation data at the time of enrollment—the visit at which BMD was first measured within the study period. Patients in this cohort typically attend routine clinical visits every 6–12 months as part of standard DMD care; however, for the purpose of this cross-sectional analysis, only data from this enrollment (baseline) visit were used.

#### 2.2.2 Treatment regimens and biochemical markers

All patients were on glucocorticoid therapy, most started on prednisone at 0.5–0.75 mg/kg/day administered as a daily regimen. Along with this, we routinely prescribed vitamin D supplements (800–1,200 IU/day) and elemental calcium (400 mg/day).

Serum levels of 25-hydroxyvitamin D [25(OH)D], calcium, phosphorus, P1NP, β-CTX, and PTH were measured. Myoglobin and creatine kinase were also recorded as markers of muscle injury. Serum 25(OH)D was analyzed using electrochemiluminescence (ECLIA) on a COBAS 411 analyzer (Roche Diagnostics).

#### 2.2.3 Body composition

Skeletal muscle mass and body fat percentage were measured using bioelectrical impedance analysis (BIA; InBody 770, InBody Co., Seoul). All measurements were performed after fasting for at least 8 h with patients wearing lightweight clothing, following the manufacturer's standardized protocol. Importantly, BIA for assessing skeletal muscle mass and body fat percentage has not been formally validated as an outcome measure in DMD populations; therefore, these measurements should be interpreted with caution.

#### 2.2.4 Functional assessment and rehabilitation

Motor function was evaluated using the Motor Function Measure 32 (MFM32), a scale specifically designed for neuromuscular diseases. The scale has 32 items across three functional domains, scored from 0 to 3, with a maximum total of 96 points; higher scores indicate better function. Assessments were performed by trained rehabilitation professionals following a standardized protocol. The Chinese version of MFM32 has been validated in pediatric DMD, with a Cronbach's *α* of 0.92 and test-retest reliability of 0.88.

Regular rehabilitation was defined as participation in structured, protocol-based rehab including joint mobility, strength, balance, gait, and functional training for at least 3 sessions per week, at least 30 min per session, documented history of at least 6 months before BMD testing. Attendance (at least 80%) was confirmed from clinical records and caregiver reports. Patients not meeting these criteria were classified as non-regular rehabilitation participants.

#### 2.2.5 BMD Z-score measurement

BMD was measured at the distal tibia using an EXA-3000 bone densitometer (OsteoSys, Seoul). Results are reported as areal BMD (g/cm²) and Z-scores, calculated using Chinese pediatric reference data adjusted for age and sex.

The distal tibia was selected for its clinical relevance in DMD. As a major weight-bearing site, this region is particularly vulnerable to disease progression and glucocorticoid-induced bone loss (13, 15). Trabecular bone at the distal tibia declines rapidly after loss of ambulation, and this site is commonly affected by fragility fractures in patients with DMD (16–18). In patients with scoliosis or spinal instrumentation, lumbar spine DXA may be unreliable, thus, distal tibia BMD serves as a practical alternative for routine bone health assessment in clinical practice.

Low BMD was defined as Z-score ≤ −2.0, in accordance with International Society for Clinical Densitometry (ISCD) pediatric guidelines.

### 2.3 Statistical analysis

Statistical analyses were conducted using SPSS 26.0 (IBM Corporation, Armonk, NY, USA). Continuous variables were presented as mean ± standard deviation, while categorical variables were expressed as frequencies and percentages. Between-group comparisons were performed using the independent *t*-test or Mann–Whitney *U*-test for continuous variables, and the chi-square test or Fisher's exact test for categorical variables as appropriate.

Variables with a *P* value less than 0.10 in univariate logistic regression were considered candidate predictors. Prior to multivariable modeling, multicollinearity among the candidate variables was assessed using two complementary approaches: pairwise Spearman rank correlation coefficients (|ρ| > 0.70 was considered indicative of substantial collinearity) and variance inflation factors (VIF) computed from ordinary least-squares auxiliary regressions with an intercept term (VIF > 10 was considered indicative of multicollinearity). Variables were excluded based on three criteria: (1) for each cluster of highly intercorrelated variables, one representative was retained; (2) variables with limited clinical relevance or unstable measurement properties were excluded on clinical grounds; and (3) variables with weaker univariate associations and no independent clinical contribution beyond the retained variables were excluded. The remaining variables were entered simultaneously into a fixed-covariate multivariable logistic regression model. Model goodness-of-fit was evaluated using the Hosmer-Lemeshow test, and overall model performance was assessed using the likelihood ratio test (LRT), Nagelkerke R^2^, and the area under the receiver operating characteristic curve (AUC).

Given that the missing rate of key variables including bone mineral density, rehabilitation status and glucocorticoid exposure history were less than 1%, complete-case analysis was adopted to handle missing data. After multicollinearity screening, five variables—age, BMI, MFM total score, ambulatory status, and regular rehabilitation—were entered simultaneously into a single fixed-covariate multivariable logistic regression model. This approach was chosen over stepwise selection to provide stable, reproducible estimates that are not influenced by variable-entry order. The analysis included 191 patients (88.8%) with complete data for all five variables and the outcome. A two-tailed *P* value < 0.05 was defined as statistically significant. Subgroup analyses by age group (<10 years and ≥10 years) and ambulatory status were performed using the same fixed-covariate Enter method, with the same five variables entered simultaneously, to explore the consistency of the association of regular rehabilitation with BMD across clinically relevant subgroups. Given the reduced sample sizes in these subgroups, the number of covariates was kept identical to the primary model to avoid overfitting.

Mediation analysis was conducted using bootstrap resampling (5,000 iterations) to quantify the indirect effect of muscle function on the association between regular rehabilitation and BMD.

## 3 Results

### 3.1 Baseline characteristics

**Figure 2:**
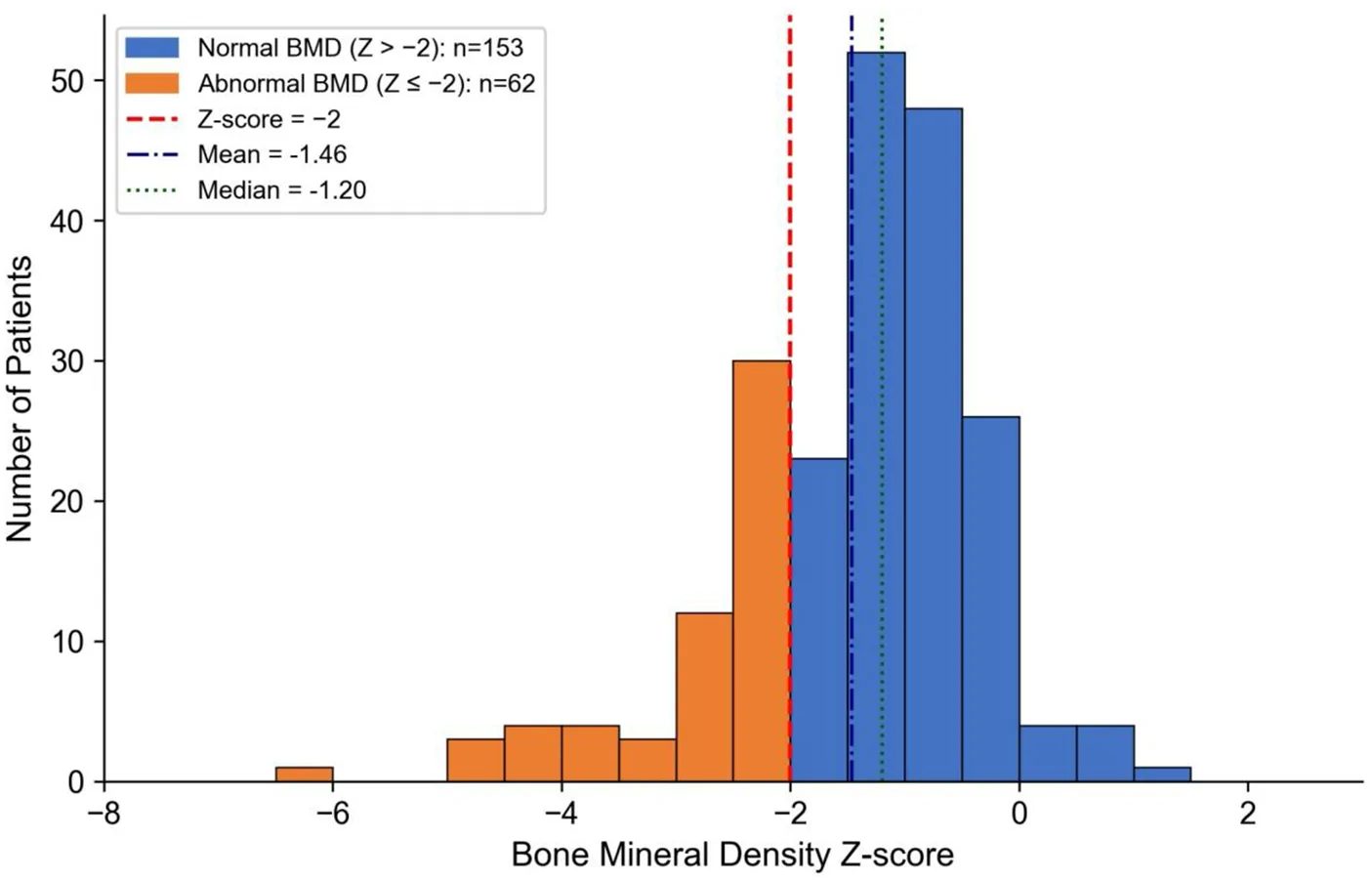
Distribution of bone mineral density Z-scores in 215 boys with duchenne muscular dystrophy.

In this cohort of 215 boys with DMD, BMD Z-scores ranged from −6.50 to +1.40, with a mean of −1.46 ± 1.10 SD. The median BMD Z-score was −1.20 (IQR: −2.10 to −0.80). Abnormal BMD (Z-score ≤ −2.0) was identified in 62 patients (28.8%), while 153 patients (71.2%) had normal BMD (Z-score > −2.0). The distribution of BMD Z-scores is shown in Figure 2.

Univariate analyses were performed to compare differences in clinical variables between patients with normal and abnormal bone mineral density (BMD), as shown in Table 1.

**Table 1:** Baseline clinical characteristics of DMD patients by BMD status.

Variable	Normal BMD (*n* = 153)	Abnormal BMD (*n* = 62)	*P* value
Age, months	80.00 (60.00–104.00)/84.08 ± 27.17	131.00 (119.00–156.00)/133.18 ± 29.03	<0.001
Age at diagnosis, months	42.00 (24.00–60.00)/45.80 ± 25.73	48.00 (36.00–76.25)/56.50 ± 29.76	0.019
Steroid use duration, months	9.00 (0.00–29.00)/20.06 ± 25.84	49.50 (18.75–79.50)/53.03 ± 38.02	<0.001
BMI, kg/m^2^	16.24 (14.86–18.71)/17.00 ± 3.05	19.50 (15.66–23.34)/19.66 ± 4.78	<0.001
Skeletal muscle mass, kg	7.60 (6.10–9.35)/7.97 ± 2.38	10.90 (8.80–12.22)/10.83 ± 2.90	<0.001
Body fat percentage, %	20.00 (13.20–31.10)/22.42 ± 11.58	35.20 (25.70–45.22)/35.04 ± 13.01	<0.001
MFM total score	0.81 (0.77–0.89)/0.82 ± 0.09	0.71 (0.56–0.78)/0.66 ± 0.18	<0.001
Trunk MRC score	0.70 (0.65–0.76)/0.71 ± 0.10	0.60 (0.50–0.65)/0.60 ± 0.12	<0.001
Upper limb MRC, %	0.84 (0.80–0.96)/0.86 ± 0.12	0.76 (0.60–0.80)/0.71 ± 0.16	<0.001
Lower limb MRC, %	0.80 (0.71–0.89)/0.79 ± 0.11	0.63 (0.54–0.71)/0.63 ± 0.15	<0.001
D1 (standing/transfer) score	0.64 (0.51–0.77)/0.64 ± 0.19	0.33 (0.08–0.56)/0.34 ± 0.28	<0.001
D2 (trunk/proximal) score	0.97 (0.94–0.97)/0.96 ± 0.03	0.94 (0.86–0.97)/0.87 ± 0.18	<0.001
D3 (distal) score	0.93 (0.86–1.00)/0.91 ± 0.09	0.95 (0.90–1.00)/0.92 ± 0.10	0.277
Cobb angle,°	5.00 (4.00–7.00)/6.42 ± 6.23	8.00 (5.00–14.25)/13.16 ± 16.66	0.002
Wechsler full-scale IQ	79.00 (73.00–86.00)/78.66 ± 11.90	76.00 (70.00–82.50)/76.05 ± 12.97	0.067
Ambulatory status, *n* (%)			<0.001
Yes	134 (87.6)	33 (53.2)	
No	14 (9.2)	22 (35.5)	
Missing	5 (3.3)	7 (11.3)	
Regular rehabilitation, *n* (%)			<0.001
Yes	125 (81.7)	22 (35.5)	
No	27 (17.6)	40 (64.5)	
Missing	1 (0.7)	0 (0.0)	
Gene mutation type, *n* (%)			0.178
Exon deletion	96 (62.7)	32 (51.6)	
Exon duplication	11 (7.2)	11 (17.7)	
Small variant	39 (25.5)	15 (24.2)	
Missing	7 (4.6)	4 (6.5)	
Corticosteroid type, *n* (%)			0.130
Prednisone	111 (72.5)	53 (85.5)	
Deflazacort	24 (15.7)	5 (8.1)	
Methylprednisolone	18 (11.8)	4 (6.5)	

Data are presented as median (interquartile range) [mean ± standard deviation] for continuous variables, and as number (percentage) for categorical variables. *P* values were calculated using Mann–Whitney *U*-test for continuous variables and chi-square test (or Fisher's exact test when appropriate) for categorical variables. *P* < 0.05 was considered statistically significant.

Compared to those with normal BMD, participants in the abnormal BMD group were significantly older at the time of assessment (median 131.0 vs. 80.0 months, *p* < 0.001) and had a later age at diagnosis (median 48.0 vs. 42.0 months, *p* = 0.019). The duration of glucocorticoid use was also markedly longer in the abnormal BMD group (median 49.5 vs. 9.0 months, *p* < 0.001). While the distribution of steroid types was comparable between groups (*p* = 0.130), prednisone remained the predominant agent in both (72.5% in the normal BMD group vs. 85.5% in the abnormal BMD group).

Anthropometric measures revealed that participants with abnormal BMD had significantly higher BMI (median 19.5 vs. 16.24 kg/m^2^, *p* < 0.001), greater skeletal muscle mass (median 10.9 vs. 7.6 kg, *p* < 0.001), and a higher percentage of body fat (median 35.2% vs. 20.0%, *p* < 0.001). Motor function was poorer in the abnormal BMD group, with lower MFM total score (median 0.71 vs. 0.81, *p* < 0.001) and lower MRC scores for trunk, upper limbs, and lower limbs (all *p* < 0.001). Among MFM subdomains, D1 score (standing and transfer) was notably lower in the abnormal BMD group (median 0.33 vs. 0.64, *p* < 0.001), while D3 score (distal function) was comparable between groups (*P* = 0.277). The proportion of nonambulatory participants was higher (35.5% vs. 9.2%, *p* < 0.001) in the abnormal BMD group.

Cobb angle was greater in the abnormal BMD group (median 8.0°vs. 5.0°, *P* = 0.002). Serum myoglobin and creatine kinase levels were significantly lower in this group (myoglobin: median 1,063.0 vs. 1,706.6 ng/mL; CK: median 7,883 vs. 15,360 U/L; both *P* < 0.001). In contrast, no significant differences were observed between groups for Wechsler total score, vitamin D, PTH, P1NP, β-CTX, or gene mutation type distribution (all *P* > 0.05) (Supplementary Table S1).

### 3.2 Multicollinearity screening of candidate variables

Prior to multivariable modeling, we assessed multicollinearity among the 18 candidate predictors identified through univariate logistic regression (*P* < 0.10). Two complementary approaches were used: pairwise Spearman rank correlation coefficients (*ρ*) and variance inflation factors (VIF) computed from ordinary least-squares auxiliary regressions among the 18 predictors.

Fourteen variable pairs exhibited strong pairwise correlation (|*ρ*| > 0.70), reflecting severe redundancy among age-related indicators (age, steroid duration, skeletal muscle mass, and body fat percentage), motor function measurements (MFM total score, D1, D2, trunk MRC, upper limb MRC, and lower limb MRC), and body composition parameters (BMI and body fat percentage) (Table 2). VIF analysis based on the 139 patients with complete data for all 18 variables confirmed severe multicollinearity: 4 variables had VIF > 10—age (13.32), MFM total score (104.24), D1 stand/transfer (65.46), and D2 trunk/proximal (16.34)—indicating severe multicollinearity driven by redundant subscale and body composition indicators.

**Table 2:** Variable pairs with strong pairwise spearman rank correlation (|*ρ*| > 0.70) among the 18 candidate predictors.

**Variable 1**	**Variable 2**	**Spearman *ρ***	**Variable retained**
Age (months)	Steroid duration (months)	0.768	Age
Age (months)	Skeletal muscle mass (kg)	0.805	Age
Age (months)	Body fat percentage (%)	0.716	Age
BMI (kg/m²)	Body fat percentage (%)	0.805	BMI
MFM total score (0–100)	Trunk MRC	0.768	MFM total score
MFM total score (0–100)	Upper limb MRC (%)	0.750	MFM total score
MFM total score (0–100)	Lower limb MRC (%)	0.825	MFM total score
MFM total score (0–100)	D1 (stand/transfer)	0.965	MFM total score
Trunk MRC	Upper limb MRC (%)	0.705	MFM total score
Trunk MRC	Lower limb MRC (%)	0.759	MFM total score
Trunk MRC	D1 (stand/transfer)	0.764	MFM total score
Upper limb MRC (%)	Lower limb MRC (%)	0.819	MFM total score
Upper limb MRC (%)	D1 (stand/transfer)	0.752	MFM total score
Lower limb MRC (%)	D1 (stand/transfer)	0.882	MFM total score

All *P* values < 0.001 (not shown). VIF values were computed from 139 patients with complete data for all 18 candidate variables using ordinary least-squares auxiliary regressions with an intercept term [consistent with standard practice, e.g., R car::vif()]. Of the 18 candidate variables, 4 had VIF > 10 in the full 18-variable analysis: age (13.32), MFM total score (104.24), D1 stand/transfer (65.46), and D2 trunk/proximal (16.34); 14 pairs showed |*ρ*| > 0.70. A total of 13 variables were excluded: 11 due to multicollinearity (steroid duration, skeletal muscle mass, body fat percentage, trunk MRC, upper limb MRC, lower limb MRC, D1 stand/transfer, D2 trunk/proximal, age at diagnosis, Wechsler IQ, myoglobin) and 2 (creatine kinase and gene mutation type) based on clinical judgment. The remaining 5 variables were entered into the final multivariable model. MFM, Motor Function Measure; MRC, Medical Research Council; BMI, body mass index; D1, stand/transfer dimension; D2, trunk/proximal dimension; VIF, variance inflation factor.

Based on these diagnostics, 13 variables were excluded before multivariable regression according to three principles. First, for each cluster of highly intercorrelated variables, one representative was retained: age was preferred over steroid duration (*ρ* = 0.768), skeletal muscle mass (*ρ* = 0.805), and body fat percentage (*ρ* = 0.716), as age is the most fundamental time-varying confounder in DMD disease progression; MFM total score was retained over its sub-domain scores (D1: *ρ* = 0.965; D2: VIF = 16.34, indicating redundancy with the MFM subscale cluster) and regional MRC measures (trunk: *ρ* = 0.768; upper limb: *ρ* = 0.750; lower limb: *ρ* = 0.825), as it provides a single comprehensive summary of gross motor function. Second, two variables were excluded on clinical grounds: creatine kinase, whose serum levels fluctuate markedly with recent physical activity and do not reliably reflect disease stage; and gene mutation type, which creates highly heterogeneous subgroups and has shown no consistent predictive value for BMD outcomes in prior literature. Third, age at diagnosis, Wechsler IQ, myoglobin, and Cobb angle were excluded due to weaker univariate associations and lack of independent clinical contribution beyond the retained variables.

The remaining five variables — age, BMI, MFM total score, ambulatory status, and regular rehabilitation — were entered simultaneously into the multivariable logistic regression model. In this reduced model (using the 191 patients with complete data for all five variables plus the outcome), VIF values computed with the intercept term ranged from 1.26 to 2.58, confirming the absence of clinically important multicollinearity.

### 3.3 Independent predictors of abnormal BMD

A total of 18 candidate variables met the threshold of *P* < 0.10 in univariate logistic regression and were considered for multivariable analysis. Following multicollinearity screening (Section 3.2), five variables were retained for the final model: age, BMI, MFM total score, ambulatory status, and regular rehabilitation. These were entered simultaneously into a fixed-covariate multivariable logistic regression model using 191 patients (88.8%) with complete data for all five variables and the outcome. All VIF values in the final model (computed with the intercept term) were below 3.0 (range: 1.26–2.58), indicating no clinically important multicollinearity.

Multivariable logistic regression revealed two independent predictors of abnormal BMD (Table 3). Age was associated with a significantly increased risk (adjusted OR = 1.0533 per month, 95% CI 1.0318–1.0752, *P* < 0.001), consistent with progressive BMD deterioration as DMD advances. Regular rehabilitation was an independent protective factor (adjusted OR = 0.2369, 95% CI 0.0935–0.6001, *P* = 0.002), corresponding to a 76.3% reduction in the odds of abnormal BMD among boys who received regular rehabilitation compared with those who did not. BMI (adjusted OR = 0.8876, *P* = 0.099), MFM total score (adjusted OR = 0.9996, *P* = 0.135), and ambulatory status (adjusted OR = 0.9570, *P* = 0.962) were not independently associated with abnormal BMD after adjustment for the other covariates. The model demonstrated excellent discrimination (AUC = 0.906) and good calibration (Hosmer–Lemeshow *P* = 0.549), with Nagelkerke *R*^2^ = 0.591 (Table 3).

**Table 3:** Univariate and multivariable logistic regression analysis of factors associated with abnormal bone mineral density in boys with Duchenne muscular dystrophy.

**Variable**	**Univariable**			**Multivariable**			**Sig.**
	**OR**	**95% CI**	***P* value**	**aOR**	**95% CI**	***P* value**	
Age (months)	1.0599	1.0429–1.0772	<0.001	1.0533	1.0318–1.0752	<0.001	***
BMI (kg/m²)	1.1978	1.1032–1.3005	<0.001	0.8876	0.7703–1.0228	0.099	
MFM total score (0–100)	0.9129	0.8840–0.9428	<0.001	0.9996	0.9991–1.0001	0.135	
Ambulatory status	0.0560	0.0197–0.1588	<0.001	0.9570	0.1594–5.7445	0.962	
Regular rehabilitation	0.1188	0.0610–0.2313	<0.001	0.2369	0.0935–0.6001	0.002	**

Dependent variable: BMD abnormality (1 = abnormal, 0 = normal). Unadjusted ORs were derived from univariable logistic regression; adjusted ORs (aOR) were derived from a fixed-covariate multivariable logistic regression model including all 5 variables simultaneously. Model fit: Likelihood ratio *χ*^2^ = 101.85 (df = 5, *P* < 0.001); Nagelkerke *R*^2^ = 0.591; Hosmer–Lemeshow *P* = 0.549; AUC = 0.906; AIC = 139.47. Analysis included 191 patients (88.8%) with complete data for all 5 variables. All VIF values in the final model (computed with intercept term) were <3.0 (range: 1.26–2.58). Significance codes: ***P < 0.001; **P < 0.01. OR, odds ratio; aOR, adjusted odds ratio; CI, confidence interval; BMI, body mass index; MFM, Motor Function Measure; VIF, variance inflation factor.

Notably, steroid duration (unadjusted OR = 1.031) and body fat percentage (unadjusted OR = 1.081) were significant in univariate analysis but were excluded from the multivariable model owing to high collinearity with age (*ρ* = 0.768 and *ρ* = 0.716, respectively), suggesting that their effects on BMD are largely mediated through age-related disease progression. Similarly, ambulatory status, which was highly significant in univariate analysis (unadjusted OR = 0.056, *P* < 0.001), lost its independent effect after adjusting for age (adjusted OR = 0.957, *P* = 0.962), indicating that the apparent protective effect of ambulatory ability is substantially confounded by, or mediated through, the trajectory of age-related disease.

### 3.4 Subgroup analysis: consistency of the association of regular rehabilitation

1. Stratification by Age
  - **10 years group (*n*** **=** **151, 18 cases of abnormal BMD, 11.9%):** Univariate analysis identified 15 candidate variables (*P* < 0.10). After fixed-covariate multivariate logistic regression, regular rehabilitation remained an independent associated factor against abnormal BMD (OR = 0.162, 95% CI: 0.035–0.751, *P* = 0.020), corresponding to an approximately 84% risk reduction in younger children.
  - **≥10 years group (*n*** **=** **64, 44 cases of abnormal BMD, 68.8%):** Thirteen candidate variables were identified by univariate screening. Regular rehabilitation again demonstrated a significant favorable association (OR = 0.143, 95% CI: 0.041–0.502, *P* = 0.002). However, due to collinearity with motor function-related measures (total MFM score, MRC muscle strength scores), it was not retained as an independent predictor in the fixed-covariate model. The markedly higher prevalence of abnormal BMD in this subgroup (68.8%) suggests that disease progression-related motor function decline may be the predominant determinant of bone health in older children.
2. Stratification by Ambulation Status
  - **Ambulatory group (*n*** **=** **167, 33 cases of abnormal BMD, 19.8%):** Univariate analysis identified 14 candidate variables (*P* < 0.10). Fixed-covariate multivariate regression confirmed **regular rehabilitation** as an independent associated factor against abnormal BMD (OR = 0.305, 95% CI: 0.102–0.914, *P* = 0.034), corresponding to an approximately 70% risk reduction in ambulatory children receiving regular rehabilitation compared to those with irregular rehabilitation.
  - **Non-ambulatory group (*n*** **=** **36, 22 cases of abnormal BMD, 61.1%):** Fourteen candidate variables were identified by univariate screening, and regular rehabilitation demonstrated a strong favorable association (OR = 0.093, 95% CI: 0.024–0.363, *P* < 0.001). However, due to the limited effective sample size (approximately 22 cases), the fixed-covariate model exhibited compromised stability, and regular rehabilitation was not retained in the final model.

**Figure 3:**
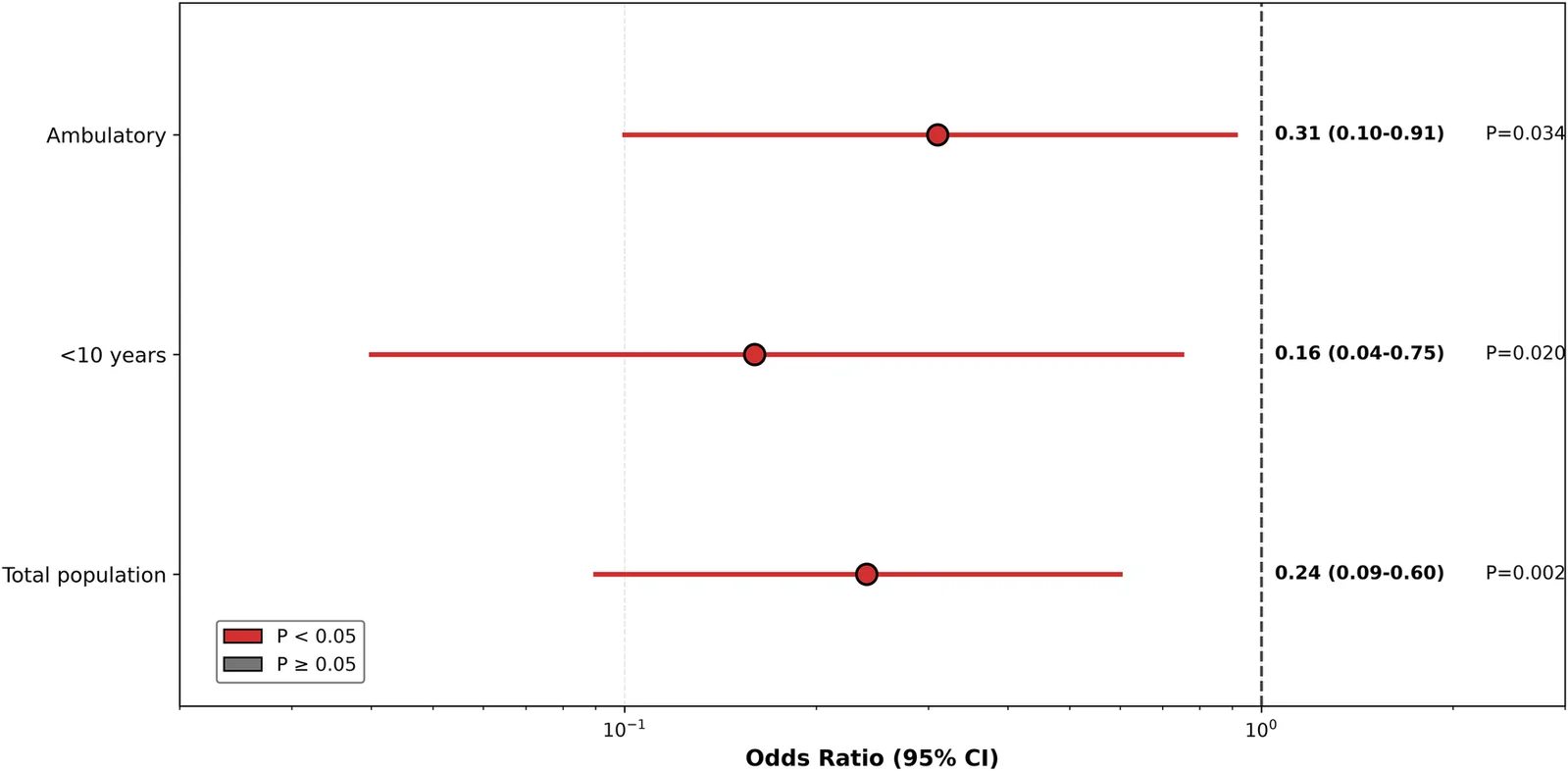
Forest plot of subgroup analysis for the association of regular rehabilitation with abnormal bone mineral density in boys with duchenne muscular dystrophy. *X*-axis shows odds ratios (OR) and 95% confidence intervals (CI) on a log scale. OR < 1 indicates a favorable association of regular rehabilitation; the vertical dashed line on the right represents OR = 1 (null effect). Red markers indicate *P* < 0.05 (statistically significant). Total population: OR = 0.237 (95%CI: 0.094–0.600, *P* = 0.002); <10 years subgroup: OR = 0.162 (95%CI: 0.04–0.75, *P* = 0.020); Ambulatory subgroup: OR = 0.305 (95%CI: 0.10–0.91, *P* = 0.034).

In summary, a significant inverse association between regular rehabilitation and abnormal BMD was observed in the total population, children <10 years subgroup, and ambulatory subgroup, with all OR values <1 (overall OR = 0.237; OR = 0.162 for <10 years; OR = 0.305 for ambulatory). The direction of the association was highly consistent across subgroups (see Figure 3), supporting regular rehabilitation as a robust independent associated factor for BMD in boys with DMD.

### 3.5 Interaction test and mediation analysis

#### 3.5.1 Test of interaction effect:moderating role of age in the association of regular rehabilitation

**Figure 4:**
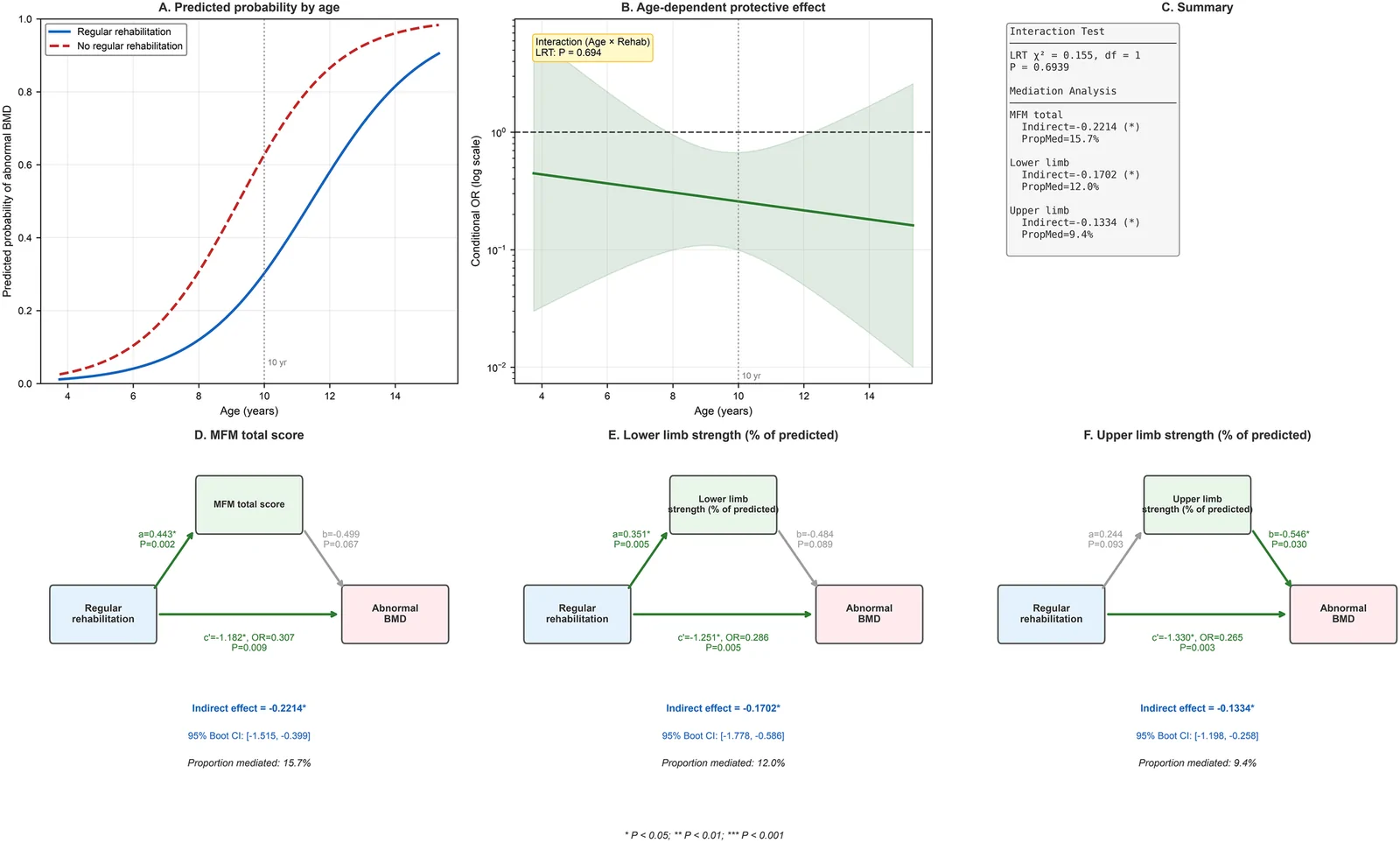
Interaction effect of age × rehabilitation and mediation analysis of muscle function. **(A)** Predicted probability of abnormal BMD by age and rehabilitation status; **(B)** Conditional favorable association of regular rehabilitation on abnormal BMD across ages (OR, log scale); **(C)** Summary of interaction test and mediation analysis. Likelihood ratio test (LRT) showed no significant interaction between age and rehabilitation (*P* = 0.6939); **(D–F)** Mediation path diagrams demonstrating that regular rehabilitation was associated with lower odds of abnormal BMD via total MFM score, lower limb strength (% of predicted), and upper limb strength (% of predicted), with mediated proportions of 15.7%, 12.0%, and 9.4%, respectively.

To further assess whether age moderates the association of regular rehabilitation, an “age × regular rehabilitation interaction term” (all variables were standardized) was added to the multivariate Logistic regression model. The likelihood ratio test (LRT) comparing the interaction model (Model B) with the main-effects model (Model A) yielded (X^2^ = 0.155), df = 1, *P* = 0.6939; the Wald test for the interaction term showed OR = 0.771 (95% CI: 0.207–2.872, *P* = 0.698) (see Figures 4A–C).

These results indicate that there is no statistically significant interaction between age and regular rehabilitation—that is, the association of regular rehabilitation on BMD does not change significantly with increasing age. This finding further supports the robustness of the favorable association of regular rehabilitation.

#### 3.5.2 Mediation analysis:mediation effect of muscle function on the association of regular rehabilitation with abnormal BMD

Mediation analysis revealed that regular rehabilitation was associated with better BMD indirectly through improving muscle function (see Figures 4D–F,). Regular rehabilitation was positively associated with total MFM score, lower limb strength (% of predicted), and upper limb strength (% of predicted) (*β* = 0.244–0.443, *P* < 0.05 or marginally significant). All muscle function measures were inversely associated with abnormal BMD, with upper limb strength showing the strongest significance (OR = 0.579, *P* = 0.030).

Indirect effects of all three mediators were significant (Bootstrap 95%CI did not include 0), with mediated proportions of 15.7% (total MFM), 12.0% (lower limb strength), and 9.4% (upper limb strength). After controlling for muscle function, the association of regular rehabilitation remained significant (OR = 0.265–0.307, *P* < 0.01), indicating additional independent mechanisms. In summary, muscle function mediated 9.4%–15.7% of the association between regular rehabilitation and BMD, supporting the pathway “Regular rehabilitation → Muscle function → .”(see Figure 4). Given the cross-sectional design of this study, the temporal relationship between rehabilitation training and muscle strength improvement cannot be established. This mediating pathway therefore represents only a hypothetical mechanism and requires validation in prospective studies.

## 4 Discussion

In this retrospective study of 215 boys with DMD, we found that 28.8% of patients had abnormal bone mineral density (BMD), with age being the strongest independent risk factor (adjusted OR = 1.0533 per month, *P* < 0.001), while regular rehabilitation, a modifiable clinical factor, was associated with a lower risk of abnormal BMD (approximately 76.3% lower; adjusted OR = 0.2369, *P* = 0.002); this beneficial association was partially mediated through better muscle function. These findings provide important evidence for bone health management in DMD.

### 4.1 Prevalence of abnormal BMD

The 28.8% prevalence of abnormal BMD in our cohort falls within the reported range (21%–45%) (19). Reported rates vary across studies due to differences in patient age composition, measurement sites, vitamin D supplementation practices, and geographic factors. For instance, Suthar et al. (20) found 57% in an Indian cohort with a 89.5% vitamin D deficiency rate, as vitamin D status is a critical determinant of bone health (20). In contrast, Wang et al. (21) recently reported 31.3% in 348 children aged 2–12 years, closer to our finding. Our cohort's broader age span (3–18 years) provides a more comprehensive picture of BMD burden across childhood and adolescence (22).

### 4.2 Age as the dominant risk factor

Our study identified age as the strongest independent risk factor for abnormal BMD in multivariable logistic regression (adjusted OR = 1.0533 per month). As patients age, they experience prolonged cumulative glucocorticoid exposure, progressive deterioration of motor function, reduced ambulation, and increased adiposity, these factors collectively contribute to bone demineralization (22, 23). While, age itself is not a causal factor, it is essentially a surrogate marker that captures the cumulative effects of multiple modifiable risk factors over time. This interpretation is supported by the strong correlations between age and both glucocorticoid duration (*ρ* = 0.768) and body fat percentage (*ρ* = 0.716), indicating that age predominantly reflects the integrated burden of disease progression and treatment exposure.

Previous studies have reported similar findings. Ma et al. demonstrated that the time to first fracture in DMD patients is strongly age-dependent, with most fractures occurring after 10 years of age (24). Similarly, Bianchi et al. reported that BMD abnormalities become more prevalent with advancing age, particularly during the second decade of life (19). However, these studies did not explore the risk factors behind age. Although age-related changes are irreversible, optimizing glucocorticoid treatment regimens, controlling body fat accumulation, and preserving motor function may potentially delay age-associated declines in BMD (15, 25, 26).

### 4.3 The favorable association of regular rehabilitation

The most clinically significant finding is that regular rehabilitation was independently associated with lower risk of abnormal BMD. Correlation analysis revealed a significant association between regular rehabilitation and normal BMD (*ρ* = −0.457, *P* < 0.0001), which multivariate analysis confirmed. Rehabilitation has long been part of standard DMD care (27, 28), but its specific effect on BMD has not been quantified before—we now show it is an independent associated factor. It is worth noting that most previous studies in this area focused on glucocorticoid adverse effects (24), while the potential benefits of rehabilitation received less attention. The failure to reach statistical significance in subgroup analyses may be related to insufficient sample size and multicollinearity, rather than true effect heterogeneity.

Several mechanisms may explain this favorable association. Weight-bearing activity provides mechanical loading that stimulates osteoblasts and inhibits osteoclasts (29). Exercise also triggers hormonal changes favoring bone formation, such as increased IGF-1 and improved vitamin D metabolism (6, 30). Maintaining ambulation preserves weight-bearing status and prevents the rapid bone loss that typically follows loss of walking (31). These mechanisms are consistent with the muscle-bone relationship recognized in pediatric bone health (32). Collectively, Our findings support making regular rehabilitation a core component of DMD management for preserving skeletal health.

### 4.4 Mediation effect:exploring the association mechanism

We tested whether muscle function mediates the favorable association of rehabilitation with BMD. Correlation analysis showed that all muscle function indicators were inversely associated with abnormal BMD: lower limb strength (*ρ* = −0.494), D1 score (*ρ* = −0.466), upper limb strength (*ρ* = −0.425), trunk strength (*ρ* = −0.409), and total MFM score (*ρ* = −0.398). Mediation analysis indicated that these parameters mediated 9.4–15.7% of the association between regular rehabilitation and BMD, supporting a potential pathway whereby regular rehabilitation was associated with higher muscle function, which in turn was related to better BMD.

This is the first study to demonstrate the mediating role of muscle function in DMD. Earlier work established that muscle function correlates with BMD. Bianchi et al. found BMD was positively associated with muscle strength in DMD patients (31), and other studies showed MFM scores correlate with BMD. Joseph et al. recently showed that losing ambulation leads to dramatic trabecular bone loss in boys with DMD (15), emphasizing how important it is to preserve muscle function. We extended this by showing muscle function explains only part of rehabilitation's benefit (9.4%–15.7%)—other pathways are involved.

These findings align with the muscle-bone axis framework (33), which posits that muscle and bone are functionally coupled, muscle affects bone through several routes (34). Muscle contraction produces mechanical loading, which is essential for maintaining bone density (29). Muscles also secrete myokines that influence bone metabolism (32). And ambulation keeps the skeleton loaded; when walking stops, BMD drops quickly (15). The direct effect of rehabilitation remained significant (OR = 0.265–0.307), suggesting neuroendocrine modulation, better tissue perfusion, and improved metabolism also play a role, these need further investigation.

Subgroup analysis showed consistent benefits across age groups and ambulation status, with no significant interaction between age and rehabilitation (*P* = 0.694). Bone protection persists throughout the disease course, so physical therapy should continue even after ambulation is lost.

### 4.5 Comparison with other risk factors

Beyond age and regular rehabilitation, univariate analysis identified several other clinical variables associated with abnormal BMD. Longer glucocorticoid treatment duration (OR = 1.031, *P* < 0.001) and higher body fat percentage (OR = 1.081, *P* < 0.001) were significant risk factors in univariate analysis. However, both were excluded from the final multivariate model due to high collinearity with age (*ρ* = 0.768 and *ρ* = 0.716, respectively), suggesting that their effects on BMD are largely mediated through the aging process.

These findings align with prior research. A 2023 systematic review confirmed that longer glucocorticoid exposure is associated with lower BMD in DMD patients (22). The relationship between glucocorticoids and body composition is also well documented—these agents promote adipogenesis while inhibiting myogenesis, leading to increased fat mass in DMD (35, 36).

The association between higher BMI and abnormal BMD seems counterintuitive, as increased body weight typically protects bone through greater mechanical loading. In DMD, however, this paradox can be partly explained by the dual effects of glucocorticoids: they simultaneously promote adipogenesis and weight gain (increasing BMI) while suppressing osteoblast activity and enhancing osteocyte apoptosis (reducing BMD) (37). Thus, higher BMI in DMD patients on glucocorticoids may reflect fat mass rather than lean mass, and the same treatment that increases BMI also directly harms bone. This is consistent with our data showing that patients with abnormal BMD had significantly higher body fat percentage (median 35.2% vs. 20.0%, *P* < 0.001) and longer steroid duration (median 49.5 vs. 9.0 months, *P* < 0.001). Similar findings have been reported in other DMD cohorts (9). Excess adipose tissue secretes inflammatory cytokines (such as IL-6 and TNF-α) that can adversely affect bone remodeling (38).

Beyond glucocorticoid-related factors, we also observed differences in laboratory markers that reflect disease stage. Serum creatine kinase (CK) levels were significantly lower in patients with abnormal BMD (*P* < 0.001)—a finding that likely reflects more advanced disease (39). As muscle degeneration progresses, fewer intact muscle fibers remain, reducing CK release into circulation. This pattern is consistent with the natural history of DMD, where CK levels tend to decline as muscle mass decreases over time (13). Similarly, lower serum myoglobin in the abnormal BMD group (*P* < 0.001) may indicate compromised muscle mass and function, further underscoring the link between muscle deterioration and bone loss.

### 4.6 Clinical implications and practical recommendations

Our findings have important clinical implications for DMD care. To the best of our knowledge, the present study is the first to demonstrate that regular rehabilitation is independently associated with a lower risk of abnormal BMD in patients with DMD, corresponding to an approximately 76.3% risk reduction after adjustment for age, ambulatory status, and other confounders. Although rehabilitation has long been part of standard DMD care (27, 40), evidence from multivariate analysis quantifying its independent association with better BMD remains limited. Our study addresses this gap.

We also examined whether muscle function mediates this bone-health beneficial association. Muscle parameters mediated 9.4%–15.7% of the effect, supporting the pathway: regular rehabilitation → better muscle function → better BMD (41).

The importance of preserving muscle function is underscored by our finding that all muscle function indicators, including lower limb strength, upper limb strength, trunk strength, and MFM score, were significantly associated with BMD (|*ρ*| = 0.398–0.494), with effect sizes second only to age. This fits with the muscle-bone axis, which posits that muscle contraction provides mechanical stimulation essential for bone remodeling and that maintaining muscle mass helps protect the skeleton (41, 42). Given that rehabilitation can preserve or improve muscle function, it may in turn help maintain BMD through this pathway.

In addition to our main findings, several clinical recommendations emerge from this study. Routine BMD monitoring should be considered in all DMD patients, with closer surveillance for those at highest risk—patients aged 10 and older, those on long-term glucocorticoids, and those with higher body fat percentage warrant particular attention (27, 40). Regular rehabilitation should be a core component of standard DMD care. The benefit was evident across all age subgroups, so it should continue throughout the disease course, even after ambulation is lost. A multidisciplinary approach is also essential. Close collaboration between neuromuscular specialists, bone health clinicians, rehabilitation professionals, and dietitians can lead to more holistic strategies for preserving bone health in DMD (27, 40).

Finally, careful optimization of glucocorticoid regimens still matters, even though treatment duration did not remain in the final multivariate model. Current evidence supports intermittent dosing when clinically appropriate to help reduce skeletal adverse effects (43).

### 4.7 Study limitations

This study has several strengths. We included a relatively large sample (*n* = 215), assessed physical activity, body composition, and various laboratory measures in detail, and applied rigorous statistical methods such as collinearity diagnostics, interaction tests, and mediation analysis.

Several limitations should also be acknowledged. This retrospective study demonstrates an association between regular rehabilitation and reduced risk of abnormal BMD. However, as this is a cross-sectional study, causality cannot be established. Future prospective cohort studies or randomized controlled trials are warranted to confirm the causal relationship.

As a single-center study, our findings may not generalize to other populations or settings; larger multicenter studies would help confirm these results. Selection bias is another concern given the retrospective design. Patients who engaged in regular rehabilitation may have been healthier overall or had better access to care. We also could not examine the link between low BMD and actual fracture risk, because fractures were not systematically recorded. In addition, details about rehabilitation, including frequency, intensity, and type, were not consistently documented, which may affect the precision of our findings. To further address potential selection bias, we conducted a propensity score matching (PSM) sensitivity analysis using 1:1 nearest-neighbor matching [calipe*r* = 0.2 × SD (logit PS)] with age, BMI, MFM total score, and ambulatory status as covariates. In the matched sample (46 pairs, *n* = 92), regular rehabilitation remained significantly associated with lower odds of abnormal BMD (OR = 0.353, 95% CI: 0.147–0.847, *P* = 0.020; McNemar's *P* = 0.019). Although the effect size was attenuated compared to the crude estimate (OR = 0.119), the PSM-derived OR was consistent with the primary multivariable analysis (OR = 0.236), confirming that the observed association is not entirely attributable to selection bias. Nevertheless, residual confounding from unmeasured factors cannot be excluded.

Additionally, skeletal muscle mass and body fat percentage were measured using BIA, a method that has not been validated as an outcome measure in DMD and may not accurately reflect true body composition changes in this specific population.

Although we adjusted for ambulatory status, MFM total score, and other indicators of disease severity in the multivariate model, we acknowledge that residual confounding by unmeasured disease severity factors cannot be entirely excluded in a cross-sectional design. Future longitudinal studies with detailed disease progression data would help further disentangle these complex relationships.

Even with these limitations, the main results remained robust in subgroup analyses and showed consistent patterns across both correlational and multivariate approaches. Overall, we believe these findings offer meaningful insights for the care of patients at risk of osteoporosis.

Future studies should consider prospective cohort designs to clarify the causal relationship between rehabilitation and bone health. Refining rehabilitation protocols to maximize skeletal benefit is another important direction. Additionally, given the potential impact of glucocorticoid administration on bone metabolism, future research should explore the effects of different dosing regimens on bone mineral density to provide more targeted therapeutic guidance. Researchers might also explore whether rehabilitation works synergistically with other bone-protective therapies such as bisphosphonates. Together, these efforts could further improve bone health and quality of life for individuals with DMD.

## 5 Conclusion

In this DMD cohort study, 28.8% of patients had abnormal bone mineral density. Through Spearman correlation and multivariate logistic regression analysis, we found that regular rehabilitation was independently associated with 76.3% lower risk of abnormal BMD. Age was the strongest independent risk factor (adjusted OR = 1.0533 per month, 95%CI 1.0318–1.0752, *P* < 0.001). Better muscle function was a key mediator of this favorable association. hese findings provide clinically relevant evidence to support regular rehabilitation as part of routine care for DMD patients.

## Data Availability

The raw data supporting the conclusions of this article will be made available by the authors, without undue reservation.
