# Supplementary material for: Risk factors for abnormal bone mineral density in Duchenne muscular dystrophy and the association of regular rehabilitation with bone health
Source: Front Pediatr. 2026 Aug 27;14:1840046. doi: 10.3389/fped.2026.1840046 (PMC13558212; doi:10.3389/fped.2026.1840046)
Supplement: Supplementary file 1 [file Datasheet1.docx]

**Table S1. Laboratory parameters by BMD status**

| **Variable** | **Normal BMD (n=153)** | **Abnormal BMD (n=62)** | **P value** |
| --- | --- | --- | --- |
| **Vitamin D, ng/mL** | 18.3 (13.5–22.0) /18.9 ± 7.7 | 17.7 (14.5–21.5) /18.4 ± 5.8 | 0.953 |
| **PTH, pg/mL** | 3.82 (3.20–5.10) /4.58 ± 3.02 | 3.79 (3.10–5.21) /4.17 ± 1.65 | 0.762 |
| **P1NP, ng/mL** | 385.5 (268.9–476.8) /387.4 ± 156.8 | 370.4 (261.5–473.3) /379.7 ± 173.8 | 0.593 |
| **β-CTX, ng/mL** | 0.87 (0.65–1.14) /0.93 ± 0.56 | 0.96 (0.65–1.16) /0.94 ± 0.39 | 0.522 |
| **Myoglobin, ng/mL** | 1706.6 (987.3–2556.0) /2084.3 ± 1730.0 | 1063.0 (670.0–1737.0) /1305.4 ± 883.2 | <0.001 |
| **Creatine kinase, U/L** | 15360 (10640–20897) /17555 ± 10935 | 7883 (5548–10948) /9259 ± 6877 | <0.001 |
